# Balancing at the Borderline of a Breed: A Case Study of the Hungarian Short-Haired Vizsla Dog Breed, Definition of the Breed Profile Using Simple SNP-Based Methods

**DOI:** 10.3390/genes13112022

**Published:** 2022-11-03

**Authors:** László Varga, Erika Meleg Edviné, Péter Hudák, István Anton, Nóra Pálinkás-Bodzsár, Attila Zsolnai

**Affiliations:** 1 Institute of Genetics and Biotechnology, Hungarian University of Agriculture and Life Sciences, Szent István Campus, 2100 Gödöllő, Hungary; 2 Institute for Farm Animal Gene Conservation, National Centre for Biodiversity and Gene Conservation, 2100 Gödöllő, Hungary; 3 Department of Animal Breeding, Institute of Animal Science, Hungarian University of Agriculture and Life Sciences, Kaposvár Campus, 2053 Herceghalom, Hungary

**Keywords:** breed assignment, Hungarian Short-haired Vizsla, IBS-central, PCA-distance, SNP-chip

## Abstract

The aim of this study was to determine the breed boundary of the Hungarian Short-haired Vizsla (HSV) dog breed. Seventy registered purebred HSV dogs were genotyped on approximately 145,000 SNPs. Principal Component Analysis (PCA) and Admixture analysis certified that they belong to the same population. The outer point of the breed demarcation was a single Hungarian Wire-haired Vizsla (HWV) individual, which was the closest animal genetically to the HSV population in the PCA analysis. Three programs were used for the breed assignment calculations, including the widely used GeneClass2.0 software and two additional approaches developed here: the ‘PCA-distance’ and ‘IBS-central’ methods. Both new methods calculate a single number that represents how closely a dog fits into the actual reference population. The former approach calculates this number based on the PCA distances from the median of HSV animals. The latter calculates it from identity by state (IBS) data, measuring the distance from a central animal that is the best representative of the breed. Having no mixed-breed dogs with known HSV genome proportion, admixture animals were simulated by using data of HSV and HWV individuals to calibrate the inclusion/exclusion probabilities for the assignment. The numbers generated from these relatively simple calculations can be used by breeders and clubs to keep their populations under genetic supervision.

## 1. Introduction

Currently, there exist approximately 450 dog breeds [1], mainly created during the Victorian era in the mid-19th century [2] bred for various working abilities including hunting, guarding, herding, morphometric, and behavioural standards which have become the dominant determinants of selection. “Breed-defining” phenotypic traits were under strong selection pressure, which reduced heterozygosity and initiated a fixation process on regions harbouring the genes with a major effect on these traits. Accordingly, a dog breed can be considered as a kind of homogeneous strain with special phenotypes and genomic makeup [2]. It was at this point that breeding clubs were formed, registration of pedigrees was required, and the reproductive isolation of the breeds gradually led to increased genetic differentiation [3,4], creating breed-barriers around the populations.

Now stand the fundamental questions: which dogs are in, and which dogs are out of this border? Which dogs can be assigned to breed, and which ones must be excluded? Before the advent of the genetic marker-based population analyses, the breed affiliation relied on breed specific phenotypes. Later, advanced genetic markers, including microsatellites and SNPs, and sophisticated computer programs provided an effective solution to answer this question more objectively.

The efficiency of different Bayesian- [5], frequency- [6], and genetic distance based [7] clustering methods have been surveyed in the individual assignment tests of 250 dogs using ten microsatellite markers. The Bayesian method ensured maximal success in determining the breeds of origins of these dogs. The most important factors of the clustering methods used were the genetic divergence of the reference populations (RPs), the polymorphism rate, and the number of microsatellites used in the analysis [8].

Is the genetic differentiation of different dog breeds distinct enough to determine the breeds of origin solely based on the genotypes of the individual dogs in question? According to Parker et al. [3], 414 purebred dogs belonging to 85 breeds genotyped on 96 microsatellite loci provided 99% success in the assignments.

Leroy et al. [9] used a panel of 21 microsatellites to genotype 1514 dogs belonging to 61 breeds. They applied a clustering approach, STRUCTURE [10], and a direct assignment method GeneClass2.0 [11]. Correct assignments of dogs were within 85.7–98.3% to their breed.

Berger et al. [12] used 13 highly polymorphic microsatellites for an assignment experiment, testing 392 dogs from 23 popular breeds in three European countries. Discriminant analysis of principal components yielded 97.5% assignment success, while the frequency-based approach [6] using software GeneClass2.0 [11] resulted in 87% overall correct assignments.

Breed and behavioural stereotypes were investigated by surveying owners of a large cohort of purebred and mixed-breed dogs called Darwin’s Ark [13]. Since the breed information relied on the owners’ reports or the breed registrations, it had to be genetically validated. A breed reference panel was assembled with known whole-genome information (sequencing, SNP-chip) of 101 breeds, each represented by 12 individuals, and artificially admixed individuals were created. In this work, approximately 700 SNP-s were used as genetic markers. ADMIXTURE [14] software was used to collate Darwin’s Ark and the global ancestry panel, which verified the breed content of mongrels and the genetic purity of purebreds.

Many of the canine, solely breed-assignment studies were performed by microsatellite markers in spite of the fact that SNPs have found their way to describe structure, admixture, and possible origin of the studied breeds [15,16]. More recent investigations, conducted mainly in cattle as another domestic species, demonstrated that the effectiveness of breed assignment experiments can be enhanced further by selecting only the most informative, so-called breed-informative, SNP-set [17]. Even ultra-low-density panels as low as 300 SNP-panel can be used successfully [18]. Different marker selection methods were applied in these publications: Delta statistics [19], fixation index (Fst) [20], and Principal Component Analysis (PCA) can be used alone or in combination with different breed assignment methods. Three software programs were widely used for the assignment of animals of unknown origin: (1) STRUCTURE [10], (2) GeneClass [11], and (3) ADMIXTURE [14]. These programs use Bayesian [5] and/or frequentist [6] methods. The software programs and statistical methods were used alone or in combination to get high, correct assignment rates [21,22,23].

In this investigation, we introduce two new simple breed assignment methods using the Hungarian Short-haired Vizsla: the PCA-distance and the identity by state or IBS-central. These can be used effectively in cases where breeders must decide on the inclusion of a phenotypically appropriate, but unregistered individual into the RP. To achieve these objectives, mixed animals are modelled to test and verify how different methods work, including the proposed PCA-distance and IBS-central methods. Real samples and the corresponding genotypes are used in all processes and analyses.

## 2. Materials and Methods

### 2.1. Sampled and Genotyped Animals

Blood samples were taken from pedigree-certified individuals into EDTA-coated tubes and were stored at −20 °C. Blood sampling was performed by trained veterinarians as part of a routine procedure for parentage testing, and, as such did not require ethical approval. A total of 142 animals were sampled. The sampling distribution was as follows: 70 Short-haired Hungarian Vizslas (HSV), 6 Wire-haired Hungarian Vizslas (HWV), 9 Transylvanian Hounds (TH), 9 Komondors (KOM), 27 Kuvaszs (KUV), 2 Hungarian Greyhounds (HG), 9 Mudis (MUD), 4 Pulis (PUL), and 6 Pumis (PUM).

### 2.2. Samples from Database

Twelve breeds from the Pointer-Setter clade (Table 1, BRIT, DALM, ESET, GSHP, GWHP, GORD, ISET, LMUN, SPIN, VIZS, WEIM, and WHPG), seven breeds from the Retriever clade (Table 1, CCRT, FCR, GOLD, IWSP, LAB, NEWF, and NSDT), and 5 breeds from the Spaniel clade (Table 1, ACKR, CKCS, ECKR, ESSP, and FIEL) were selected from the database [24] which contains 161 breeds.

### 2.3. Modelled Animals

To test how the proposed assignment methods work, dogs with different HSV genome proportions were needed in addition to the purebred HSVs. Genotypes of these hypothetical animals were artificially created from the actual genotype data acquired by genotyping. Admixed animals were created as follows: An ‘empty’ genome was loaded in steps from the genotypes of individuals of 24 Vizsla-related breeds (Table 1) [24] and from an HSV genotype. A total of 924 SNPs (Table S1) were used. These SNPs are in ascending order of chromosome number and position. In order to assemble the genome sequentially from 25 animals, approximately 1/25 of each genome contributes to the artificial individual. In this instance, 38−38 of the 24 breeds and 12 SNPs of the 25th GORD breed were entered into the artificial genome (Table 2). For example, the first region contains 38 SNPs from the ESSP, followed by the next region from the CKCS breed and so forth. According to this allocation, the end of one chromosome and the beginning of the next chromosome could be included in a region delimited by 38 SNPs. Following these methods, two artificial animals (Admix1 and Admix2) were created to serve as the controls in GeneClass2 runs.

For testing the ability of inclusion/exclusion of animals at different admixture levels by GeneClass2 software, PCA-distance, and IBS-central, different percentages of loci of selected HSVs were replaced by the genotypes of an HWV animal in the same manner as described above.

### 2.4. 234 K SNP-Set

SNP typing of the samples was accomplished using a chip containing the SNPs of Illumina Canine HD chip (Illumina, San Diego, CA, USA) as well as SNPs by the LUPA consortium [25,26]. Genotyping was performed by Neogen Corporation (Ayr, UK). The SNP-chip contains 234 K SNPs including the subset of markers described by Parker et al. [24].

### 2.5. Merge of Databases and Filtering for HSV-Enhanced SNP-Set

**Figure 1:**
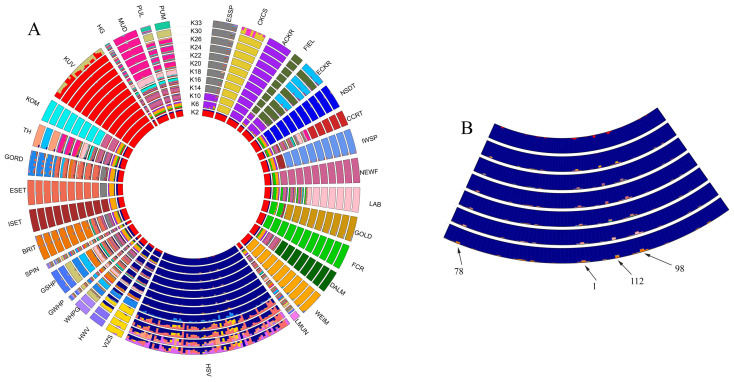
(**A**) Admixture of 33 breeds based on 145,453 SNPs. (**B**) Enlarged part of Figure 1A. For the resolution of the acronyms see Table 1 and Table 2.

**Figure 2:**
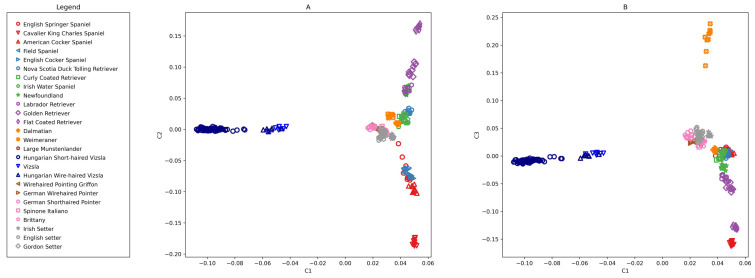
Principal component analysis of Hungarian Short-haired Vizsla, Hungarian Wire-haired Vizsla, and 24 breeds from Parker et al. [24] using 145,453 SNPs. Eigenvalues of the components are C1 = 15.731, C2 = 7.008, and C3 = 6.229. (**A**) C2 vs. C1 (**B**) C3 vs. C1.

**Figure 3:**
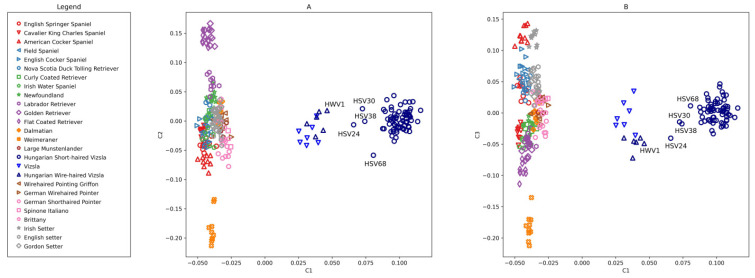
Principal component analysis of Hungarian Short-haired Vizsla, Hungarian Wire-haired Vizsla, and 24 breeds from Parker et al. [24] using 924 SNPs. Eigenvalues of the components are C1 = 106.785, C2 = 3.700, and C3 = 3.548. (**A**) C2 vs. C1 (**B**) C3 vs. C1. HSV individuals numbered 24, 30, 38, and 68 do not belong to the central part of the Hungarian Short-haired Vizsla population. HWV individual numbered 1 has been selected to denote the border of HWV.

An important aspect of this research was to compare the former version of Illumina Canine HD (174 K) genotypes of the breeds included in the database [24] with the genotypes of the Hungarian samples typed on the Canine HD (234 K) chip. The 174 K [24] and the 234 K datasets were merged and used in the Admixture (Figure 1) and PCA studies (Figure 2). In the merged dataset, only those SNPs that were present in both sets were used, which had a call rate above 0.95. After filtering, the merged dataset contained 145,453 SNPs. It was then used to search for HSV-enhanced markers. The HSV was compared to all the other breeds in the Parker et al. database [24], except the VIZS breed, by calculating the Fst values of the markers. Only those SNPs were retained from the HSV-enhanced set for further analyses, which had Fst values higher than or equal to 0.4 and were not in linkage disequilibrium (threshold = 0.5, composite haplotype method, [27]). Finally, 924 SNPs were selected into the HSV-enhanced set and were used in GeneClass2 software, PCA (Figure 3), PCA-distance, and IBS-central methods.

### 2.6. Calculated Indices and the Software Packages Applied

Call rate calculations of markers and samples as well as Fst values of the markers, were performed by the SNP & Variation Suite (SVS) program (GoldenHelix, Bozeman, MT, USA). Genome-wide pairwise IBS values were determined by both SVS and PLINK [28]. The above-mentioned matrix of pairwise IBS values in PCA was performed by SVS and PLINK to acquire the positions of the animals relative to each other.

To assess the ratio of mixed ancestry of animals, the ADMIXTURE software v.1.3 was used with the –cv option to determine the most probable cluster number (K) from the value of cross-validation error in each Ks [29]. The cross-validation was performed five times, and the algorithm was terminated when the log-likelihood increased by less than 10^−4^ between iterations. Before analysis, the alleles of the SNP loci were recoded to numerical values 1 and 2 by PLINK using the –recode12 switch as required by ADMIXTURE.

The inclusion probabilities were determined by GeneClass2 [11], and distances to reference points were determined by the PCA-distance and IBS-central methods. In GeneClass2, the computation goal was to assign individuals to a reference population by choosing Rannala & Mountain [5] criterion and the simulation algorithm of Paetkau et al. [30].

### 2.7. PCA-Distance

PCA-distance is built on the coordinates given by principal component analysis. In PCA, the animals are positioned in a three or more-dimensional space. From a reference point in that space, the standardized distance to individuals can be determined (Table 2). The reference point, defined here as the median of HSV individuals, is calculated solely from the principal component values of HSV animals. The outgroup is a single HWV individual (HWV1) being the closest to the HSV group in the PCA analysis (Figure 2 and Figure 3). This HWV individual indicates the maximum distance to the median of HSVs.

During the assignment step of a new animal into the population, the PCA coordinates of the new and all RP animals are determined as well. As a result, the PCA coordinates of individuals change at each consecutive assignment step, and the standardised distances of all animals to the actual HSV-median are recalculated.

### 2.8. IBS-Central

This model is based on genetic similarity to a central animal. The method calculates the pairwise genetic similarity matrix of the individuals in the studied population (PLINK, SVS). In that symmetric matrix, the values of the pairs remain unaffected constants between two animals during the iterative calculations. Each dog is characterised by the sum of the identity by state values in that row (IBS_sum_). The individual with the maximum sum value (IBS_max_of_sums_) is the central animal being the most similar one to all other individuals. The delta value of an animal is equal to IBS_sum_ − IBS_max_of_sums_. The delta values are normalised between 0 to 1. The 0 value belongs to the central animal, and the 1 value indicates the outgroup, the HWV1 individual.

When inserting a new animal into the population, it is sufficient to calculate the pairwise IBS values of the new individual relative to existing individuals in the population, but all IBS_sum_ values of the animals must be recalculated. An insertion can also change the identity of the central animal.

## 3. Results

### 3.1. Admixture

If the analysed populations are listed clockwise (Figure 1), the first two clades are more distant relatives of the Vizsla breed: the Spaniels and the Retrievers. The Pointer-Setter Clade varieties, including the HSV and HWV breeds, begin with the DALM and end with the GORD population. At the end of the round, the other seven breeds are found—Hungarian breeds which are not related to the Vizsla. Among the K2-33 levels, the K = 18 grouping has the lowest cross-validation error rate; this is the optimal group size. The subject of this study, the HSV group, already forms a completely homogeneous set at the K = 2 level and maintains this until K = 20. Figure 1B depicts the HSV population in more detail and highlights four individuals (HSV24, 30, 38, and 68) who are slightly different genetically from the majority of HSV animals. The HWV and VIZS groups show strong similarity. Since only the origin of HWV and HSV individuals are known to us, and not that of VIZS individuals, the reason for the genetic similarity of the populations can only be speculated. The HWV group is also similar to the HSV group. This is consistent with the history of the HWV breed since it was created by crossbreeding of the HSV and the GWHP breeds during the 1930’s [31].

There are seven Hungarian breeds unrelated to the Vizsla separated quickly and uniformly at early K values, such as the KOM and KUV groups. Some of these do not give a uniform pattern within themselves, even at K = 20, such as the HG. The PUL and PUM populations do not separate from each other, even at K = 30, reflecting the close relationship between the two species. Additionally, the MUD group displays strong similarities with the PUL and PUM groups.

For the breeds coming from Parker et al. [24], Spaniels and Retrievers at the K = 20 level are well structured except for CCRT, with some breeds showing individuals protruding from the group (ESSP and ECKR). In the Pointer-Setter group, there are breeds separated and structured to the K = 20 level, including DALM, WEIM, and ESET, and there are some that do not differ in this analysis even at K = 20, including WHPG-GWHP-GSHP-BRIT. Finally, some are not structured at all, presumably due to the small number of samples (Figure 1). For more details see Parker et al. [24].

### 3.2. PCA

#### 3.2.1. PCA on Merged Dataset, 145,453 SNPs

The PCA study with 145,453 SNPs on 26 species clearly distinguishes both the HSV group and the overlapping HWV and VIZS groups from the other species (Figure 2). Eigenvalues of axes 1 to 3 are 15.731, 7.008, and 6.229, respectively.

#### 3.2.2. PCA on HSV-Enhanced, 924 SNPs

PCA analysis was also performed on 26 breeds with the HSV-informative set containing 924 SNPs (Figure 3). The first component (eigenvalue = 106.785) separated the HSVs and closely related HWVs with much greater power than in the previous analysis (Figure 2), where the eigenvalue of the first axis was 15.731, but, at the same time, the other breeds were closer to each other. The distribution tendency of the animals was similar as seen in Figure 2, but the Vizsla populations stretched more in the height of the Y-axes. This resolution showed that four HSVs (samples 24, 30, 38, and 68) were somewhat detached from the main ‘cloud’ of this population towards HWV. The HWV and VIZS groups were closely aligned.

### 3.3. Assignments

The individuals in the RP and the individuals added in each step were random. However, they have followed the order of the animals in this database.

#### 3.3.1. GeneClass2

- Assignments of 40 HSVs

The initial number of the RP was 30 (Table 3). At each assignment step, two new animals were offered to the constantly increasing RP. There were 20 assignment steps altogether. For each step, five individuals were offered to the RP, comprised of two new HSV individuals and the following three negative controls: HWV1, being the closest to the HSV cloud on the PCA plot (Figure 3), and two artificially admixed animals (Admix1 and Admix2).

For an animal to be assigned, the GeneClass2.0 software provides an inclusion probability number ranging from zero to one. Zero is a complete exclusion, and one indicates a maximal fit into the population. As expected, GeneClass2.0 accepted all HSV individuals—being purebreds by registry and confirmed genetically by the Admixture program—and excluded all three negative controls. The negative controls always acquired zero values, while HSV samples had values from 0.112 to 0.999. Exceptions were two animals at the fourth and nineteenth entry step with 0.045 and 0.007 inclusion probabilities, HSV38 and HSV68 (Table 3).

- Assignments of diluted HSVs

The PCA analysis located the HSV45 and HSV67 animals into the centre of the HSV population distribution (Figure 2 and Figure 3, HSV45 and HSV67 are not marked), having high inclusion probabilities, 0.921 and 0.998, respectively, during GeneClass2 assignments (Table 3). Two additional animals from the periphery, HSV38 and HSV68, having low inclusion probabilities, 0.045 and 0.007, respectively, in GeneClass2 output (Table 3), were also used for creating artificially admixed animals.

The genomic proportions of these four animals were diluted/replaced by the genome of the HWV1 individual. The first column of Table 4 lists the extent of the HWV portion. In the row of 0%, the original inclusion probabilities of the four HSVs are shown. These values are slightly different from those shown in Table 3, mainly because these individuals were reassigned to 66 HSVs.

#### 3.3.2. PCA-Distance

In the first step, the RP is formed from 30 HSV individuals. A HWV1 individual sets the maximum PCA-distance value, highlighted in red. PCA-distance values above 0.6 were also marked in red. The RP in each column has a minimum value, indicated in blue, which points to the animal currently closest to the HSV-median of the PCA (Table 5).

The minimum values vary among seven individuals in a total of 21 consecutively expanding RPs (compacted dataset; Table 5, full dataset; Supplementary Table S3). Some individuals regained the lowest value twice or three times. The dynamics and influence of insertions and recalculations can be tracked by the fluctuating positions of the individuals (Figure S1). This dynamic nature could be noticed in the movement of the values above 0.6 as well (Table 5). In that table, five individuals are protruding from the main population (HSV24, 28, 30, 33, and 68). At least once, four individuals show a value higher than 0.6, which also varies with an increasing RP. The value above 0.6 is displayed more than once for HSV28 but went below 0.6 at later entry steps, never regaining its high value.

#### 3.3.3. IBS-Central

Among all 30 individuals of the starting RP, the pairwise IBS values were determined. These values are in a 30 × 30 matrix (Table S2). In the final column of a row, the values are summed. In the RP, the central animal is the one having the highest summed value, normalized of zero. As in the PCA-distance method, an outer border had to be defined, which is determined by the HWV1 individual. This dog had the smallest summed IBS value, or, in other words, it had the lowest similarity to all other HSVs. Its value is standardized to one.

The layout of Table 6 is identical to that of Table 5. Here, the blue colour specifically indicates zero, which belongs to the central animal in a particular RP. The red colour indicates the maximum value set by HWV1 and any value above 0.4.

#### 3.3.4. Standardised Values of Modelled HSVs by PCA-Distance and IBS-Central Methods

To test the functioning behaviour of the two methods presented here, the same animals (HSV38, 68, 45, 67) and their admixed versions, with 10% increments in the genomic fraction of the HWV1 (Table 7), were used as previously presented with GeneClass2 (Section 3.3.1). The PCA-distance gave 1.3–2.3-fold higher standardised distances or exclusion probabilities compared with the IBS-central method. In both approaches, the most protruding specimen, HSV68, reaches and even exceeds one in the PCA-distance at 60–80% of the HWV ratio.

## 4. Discussion

In the admixture analysis (Figure 1A), the PUM and PUL groups appeared to be very similar, as the Pumi breed was created by crossbreeding the primitive Puli with German and French terrier-type herding dogs [32]. HWV and VIZS groups showed strong similarity to each other and also to the HSV group. The HSV population formed a homogeneous subset, confirming the purebred status supported by the pedigree information provided by the breeders. Within this group, four individuals (HSV 24, 30, 38, and 68) were identified who displayed slightly different admixed patterns than those of the majority of HSV animals (Figure 1B). These peripheral animals have also been highlighted by PCA GeneClass2, PCA-distance, and IBS-central approaches.

The PCA analysis confirmed the separation of HSVs from other, related breeds both by using the merged dataset and the Parker et al. [24] dataset by using the HSV-enhanced 924 SNPs. The last set was extracted from the larger set by contrasting HSV and the remaining breeds. This smaller set, used in subsequent analyses, has larger discrimination power determined from the eigenvalue of axis one, which increased from 15.731 (Figure 2) to 106.785 (Figure 3). Since the HSV-enhanced set contains the most different loci of HSV from the other breeds, the resolution of Vizsla individuals improves, while that of the other breeds is slightly deteriorating.

The unified first step in the assignment procedures is to build an initial RP. It is unrealistic to determine the size of an RP only in a few animals, but the optimization began from a very low number of dogs in testing the PCA-distance and IBS-central methods (Figure S2). The size of the initial RP was set at 30 individuals, and its size was increased from this point.

GeneClass2 does not automatically exclude individuals that had already been included in the RP in the initial assignment steps but later appeared to be outliers. This is a cautious technical approach because such a removal is especially undesirable when the number of the RP is still low, since an animal that appears as an outlier could fit later with increased number of genotyped animals becoming available during the subsequent assignment steps. With higher RP numbers, the RP is more likely to represent the entire breed, including animals not yet genotyped and/or bred outside the country.

Assignment expectations based on the admixture analysis of 40 HSVs to the RP containing 30 HSV dogs have been confirmed by GeneClass2 (Table 3). Two animals (HSV24 and 30), which proved to be slightly different from the majority of the HSV population by admixture analysis (Figure 1B), were randomly assigned into the RP. The other two animals (HSV38 and 68), which also appeared to be more admixed in Figure 1B, had much lower inclusion probabilities than the other 38 specimens.

To test how GeneClass2 classifies differentially admixed animals, assignments of simulated HSVs and HWV animals were performed. To create initial genomes that are gradually diluted, two HSVs with low and two with high inclusion probabilities were selected based on the values of Table 3.

As expected, the inclusion probabilities declined steadily faster during the dilution of HSV genomes with HWV genomes in the case of the two peripheral animals: HSV38 and 68. These animals zeroed at 50 and 30%, respectively, while HSV45 and 67 of the central core of the HSV population, zeroed at 70 and 80% genome exchange, respectively (Table 4). In all four cases, a large decrease in the inclusion probability value was observed at 10% HWV ratio. The smallest decrease occurred in HSV67, which shows that the method detects the foreign genome fraction with good sensitivity even in a small proportion and even if this foreign proportion is coming from a very close breed.

These two newly presented assignment approaches, at the current stage, do not decide on inclusion/exclusion. Prior to each assignment step, the size of the RP should be modified based on the previous step in the same way as done here in the case of GeneClass2. If there is an animal that has a significantly different probability of belonging to the main group than the others, it will be shifted towards the periphery of the group. When the RP is large enough, individuals above the empirically set threshold can be removed after each assignment step. The inclusion/exclusion threshold may change with the increase in the RP and could be determined based on the actual data. Both the PCA-distance and IBS-central methods give values between zero and one, which can be interpreted as an inclusion/exclusion probability. That number represents the entire genome and its similarity or dissimilarity from all other animals in the population. When this number is closer to the value one, it could be interpreted that one or more of the individual’s ancestors must have belonged to a related breed. Consequently, an animal with a value above a certain threshold, which must be established on professional considerations, is not desirable to be classified in the RP. This decision process could be called “balancing at the breed boundary”.

Individuals who do not fit into the RP will drift to the periphery with this approach. If they do not shift closer to the core in the subsequent steps, they must finally be removed manually at a high RP number, when it can be assured that the individual is not one of the extreme specimens of a breed but an outlier animal.

The PCA-distance approach generates a measure of fit to the current RP by specifying the standardised distance of a given individual relative to the median value calculated from the individuals of the RP. Accordingly, it does not matter whether the PCA places the individuals in a two, three, or higher-dimensional space. This method has two notable points: the median value of HSV individuals and the cut-off/maximum value from the median of HSVs, which is designating the outer circle of the breed boundary. Above that maximum value is the territory of another breed. A single individual of the nearest breed is enough for establishing this demarcation point. Given the experience of working with HSVs, it is obvious that its closest relative breed is the HWV. Within HWV, a single individual has been chosen, HWV1, which is closest to the HSV population.

In the IBS-central approach, the most prominent representative of the actual RP of the breed is the individual whose similarity to the other individuals is the highest and, as such, is the one representative of the breed worthy for whole genome sequencing. This animal has the shortest summed distance from all individuals in a given RP.

There were four among the certified purebred HSVs who were slightly peripherical compared to the central core of the HSVs. The most distal HSV in PCA-distance and IBS-central analyses was the HSV24 individual. The two analyses differ in the assessments of HSV38. The PCA-distance displayed seven individuals to be more protruding than this individual. The IBS-central animal method compresses the main group and separates the outlier individuals more explicitly from the main group. Considering these similar results, the IBS-central method might be better suited for practical breed assignments as it distinguishes more explicitly individuals residing in the peripheral region in the breed distribution.

When comparing the results of PCA-distance and IBS-central methods (Table 5 and Table 7), both low and high values occur relatively consistently in the two analyses.

**Figure 4:**
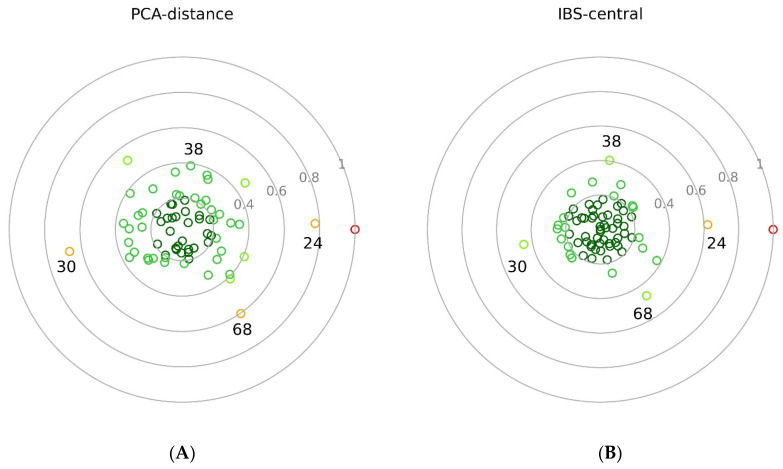
Results of PCA-distance (**A**) and IBS-central (**B**) methods. Coloured small rings are representing the individuals with different standardised values ranging from 0 to 1. Grey circles and numbers denote the distance from the zero. In the middle of the circular plots, the zero point represents the median of the HSV (**A**) and the central animal HSV51 (**B**). Animals with 0–0.2 values are coloured in deep green, 0.2–0.4 in green, 0.4–0.6 in neon green, and 0.6–08 in orange. Red represents the HWV animal at the value of 1. Animals numbered 24, 30, 38, and 68 are also HSV animals.

The values of the last columns of the two analyses (the data set of the last columns labelled by ‘70′ in Table 6 and Table 7) were also plotted on two circular plots (Figure 4). It is more appropriate to refer to them as ‘dotted balls’ since it reflects the dynamic nature of the calculations and the continuous changing of the RPs.

On both dotted balls, the HWV1 individual is at 1.0. In the PCA-distance calculation (Figure 4A), the inner two circles, 0–0.2 and 0.2–0.4, are not overcrowded, and there is no animal in the centre. In the IBS-central animal approach (Figure 4B), the data is more condensed in the inner two circles, and the HSV51 central animal is at the origo. The same two data series are also presented in tabular form (data series in the last columns labelled as ‘70′ in Table 5 and Table 6) in ascending order (Table S4).

In the PCA-distance method (Table 5), the 0.6 value itself is an arbitrarily chosen threshold, which is not the borderline of the breed. This value is selected to highlight the animals on periphery as a first trial. The positions of these animals are in good accordance with admixture, PCA, GeneClass2, and IBS-distance results. The borderline of the breed could be clarified as the number of the RP increases. In the IBS-central method, the number of highlighted animals on the periphery is lower than that of the PCA-distance method, since the overall normalised values were also lower in IBS-central. Four individuals (HSV24, 30, 38, and 68) appear to have values above 0.4 (Table 6). These animals were indicated to be slightly different by the admixture analysis based on the merged, 145,453 SNP-set (Figure 1B) and by the PCA plots (Figure 2 and Figure 3). As can be seen, the position of the central animal is changing between two individuals under the influence of consecutive assignments and recalculations. In the IBS-central results, the oscillation of the standardised distances is more attenuated than in the PCA-distance results.

To test the behaviour of these two methods, the same animals (HSV38, 68, 45, 67) and the corresponding admixed versions with 10% increments in the genomic fraction of the HWV1 (Table 7), were used as previously presented with GeneClass2 (Section 3.3.1). In the case of PCA-distance, at 60−80% HWV ratio, the mixed HSV68 specimens exceeded the 1.0 outer border of HSV set by the single HWV1 individual. Based on assumption and as seen in Figure 1, to some extent, the HSV68 may already carry an HWV or another closely related breed background, such as a breed from the Pointer-Setter clade, and thus its admixed genome became further away than the outer point, the HWV1 dog itself. Vice versa, it is noticeable that the HWV1 genome carries regions more specific to HSV due to a very small genome proportion left from the initial HSV x DWHP cross when the HWV breed creation started.

As mentioned earlier, we set PCA-distance and IBS-central initial threshold values to 0.6 and 0.4, respectively. If the breeders decide that a 20% foreign DNA ratio is acceptable, the inclusion limit into the RP could be set as 0.820 and 0.570, derived from the results of artificially mixed animals (Table 7). Based on the current image of the sampled 70 HSV animals, inclusion limits could be 0.800 (Table 5) and 0.650 (Table 6) in the cases of PCA-distance and IBS-central methods, respectively.

## 5. Conclusions

Two simple SNP-based methods were designed and presented to help breeders’ decision to assign new individuals to the reference population (RP) of the Hungarian Short-haired Vizsla. The PCA-distance method calculates the standardised distances of individuals to the median position of the breed members defined by the coordinates of Principal Component Analysis. The IBS-central method calculates standardised distances based on identity-by-state values, where the reference point is an animal who is genetically the closest to everyone in the RP. The outer border of a breed is defined by the genetically closest member of the most closely related breed: the Hungarian Wire-haired Vizsla.

We plan to genotype more animals from other breeds or species as well and to establish where the described procedures can be put into work with high confidence. From the results presented here, based on Admixture, PCA, GeneClass2, PCA-distance, and IBS-central analyses, we conclude that 70 animals for an RP are satisfactory for the Hungarian Short-haired Vizsla.

## Figures and Tables

**Table 1:** Pointer-Setter, Retriever, and Spaniel clades, breeds, and their acronyms [24].

Breed	Abrev.	Clade	Animal No.
Brittany	BRIT	Pointer-Setter	10
Dalmatian	DALM	Pointer-Setter	9
English Setter	ESET	Pointer-Setter	10
German Short-haired Pointer	GSHP	Pointer-Setter	10
German Wire-haired Pointer	GWHP	Pointer-Setter	2
Gordon Setter	GORD	Pointer-Setter	10
Irish Setter	ISET	Pointer-Setter	9
Large Munstenlander	LMUN	Pointer-Setter	3
Spinone Italiano	SPIN	Pointer-Setter	2
Vizsla	VIZS	Pointer-Setter	7
Weimaraner	WEIM	Pointer-Setter	10
Wire-haired Pointing Griffon	WHPG	Pointer-Setter	6
Curly Coated Retriever	CCRT	Retriever	6
Flat Coated Retriever	FCR	Retriever	10
Golden Retriever	GOLD	Retriever	10
Irish Water Spaniel	IWSP	Retriever	10
Labrador Retriever	LAB	Retriever	10
Newfoundland	NEWF	Retriever	10
Nova Scotia Duck Tolling Retriever	NSDT	Retriever	10
American Cocker Spaniel	ACKR	Spaniel	10
Cavalier King Charles Spaniel	CKCS	Spaniel	10
English Cocker Spaniel	ECKR	Spaniel	10
English Springer Spaniel	ESSP	Spaniel	10
Field Spaniel	FIEL	Spaniel	4

**Table 2:** Assembling the genomes of two artificially mixed animals (Admix1 and Admix2) from the HSV and HSV-related breeds.

Admixture Step	Breeds	SNP Number Added	Serial of SNP (From–To)
1	ESSP	38	1–38
2	CKCS	38	38–76
3	ACKR	38	77–114
4	FIEL	38	115–152
5	ECKR	38	153–190
6	NSDT	38	191–228
7	CCRT	38	229–265
8	IWSP	38	267–304
9	NEWF	38	305–342
10	LAB	38	343–380
11	GOLD	38	381–418
12	FCR	38	419–456
13	DALM	38	457–494
14	WEIM	38	495–532
15	LMUN	38	533–570
16	VIZS	38	571–608
17	WHPG	38	609–646
18	GWHP	38	647–694
19	GSHP	38	695–722
20	SPIN	38	723–760
21	BRIT	38	761–798
22	ISET	38	799–836
23	HSV *	38	837–874
24	ESET	38	875–912
25	GORD	12	913–924

The first column shows the genome assembly steps, the second shows the breed name abbreviations in the database [24], the third denotes the number of SNPs representing a genome region, and the fourth represents the accumulated serial number of the SNPs ordered by chromosome and position (Table S1). * denotes the step where this study’s HSV animals were included in the construction of admixed animals.

**Table 3:** Assignments of animals to the reference population (RP) by GeneClass2. Starting number of the RP is 30. Animals to be assigned are two in numbers in each step. After each step, the RP number increases by two.

Assignment Step	HSV ID	Assignment Probability
1	32	0.325
	33	0.181
2	34	0.729
	35	0.386
3	36	0.835
	37	0.286
4	38	0.045
	39	0.652
5	40	0.702
	41	0.462
6	42	0.381
	43	0.890
8	44	0.807
	45	0.921
9	46	0.888
	47	0.958
10	48	0.471
	49	0.268
11	50	0.119
	51	0.999
12	52	0.329
	53	0.178
13	54	0.199
	55	0.817
14	56	0.970
	57	0.807
15	58	0.722
	59	0.513
16	60	0.566
	61	0.643
17	62	0.555
	63	0.282
18	64	0.212
	65	0.774
19	66	0.797
	67	0.998
20	68	0.007
	69	0.118
21	70	0.644
	71	0.513

**Table 4:** Increasing the genomic fraction of four HSV individuals, with low and high assignment probability at 0% admixture, by 10–90% of the genome of HWV1 individual.

		lowP		highP	
% Admix	HWV1	HSV38	HSV68	HSV45	HSV67
0	0	0.025	0.008	0.803	0.999
10	-	0.003	0.001	0.089	0.457
20	-	0.002	0.001	0.033	0.073
30	-	0.002	0	0.009	0.028
40	-	0.001	0	0.003	0.006
50	-	0	0	0.002	0.002
60	-	0.001	0	0.001	0.002
70	-	0	0	0	0.001
80	-	0	0	0	0
90	-	0	0	0	0

**Table 5:** Results of PCA-distance method. Starting number of the RP is 30. Blue denotes the animal closest to the median of the RP. Red denotes the border defined by an HWV animal and the HSVs farthest from the median of the RP. Due to space limitations of the tables, not all animals (rows) and addition steps (columns) are presented here; full dataset is included into Supplementary Table S3.

Animal Number	30	32	34	38	40	46	50	52	56	60	64	66	68	70
ID	Distance to HSV Median													
HWV1	1.000	1.000	1.000	1.000	1.000	1.000	1.000	1.000	1.000	1.000	1.000	1.000	1.000	1.000
HSV2	0.025	0.038	0.062	0.062	0.070	0.097	0.098	0.141	0.215	0.193	0.205	0.189	0.129	0.137
HSV3	0.336	0.143	0.134	0.178	0.204	0.219	0.213	0.250	0.267	0.292	0.317	0.310	0.341	0.342
HSV4	0.307	0.160	0.079	0.047	0.070	0.063	0.062	0.029	0.033	0.027	0.039	0.050	0.208	0.214
HSV5	0.323	0.132	0.124	0.170	0.178	0.180	0.180	0.185	0.192	0.206	0.255	0.258	0.304	0.306
HSV6	0.094	0.145	0.225	0.252	0.215	0.198	0.178	0.172	0.174	0.198	0.196	0.206	0.283	0.266
HSV7	0.121	0.135	0.161	0.229	0.252	0.261	0.233	0.204	0.169	0.193	0.168	0.188	0.089	0.091
HSV8	0.307	0.292	0.217	0.132	0.190	0.200	0.206	0.216	0.243	0.321	0.254	0.262	0.278	0.276
HSV9	0.059	0.067	0.079	0.057	0.048	0.072	0.074	0.105	0.208	0.176	0.186	0.169	0.121	0.135
HSV10	0.243	0.254	0.167	0.178	0.163	0.147	0.158	0.179	0.182	0.199	0.160	0.185	0.196	0.194
HSV11	0.361	0.242	0.201	0.285	0.291	0.260	0.323	0.320	0.332	0.322	0.337	0.354	0.407	0.413
HSV12	0.143	0.066	0.225	0.208	0.217	0.162	0.145	0.148	0.148	0.175	0.156	0.196	0.220	0.222
HSV13	0.117	0.144	0.131	0.106	0.175	0.230	0.228	0.233	0.235	0.242	0.218	0.222	0.207	0.208
HSV14	0.139	0.167	0.198	0.154	0.161	0.145	0.150	0.113	0.109	0.117	0.100	0.139	0.079	0.074
HSV15	0.150	0.160	0.169	0.178	0.137	0.117	0.122	0.115	0.121	0.128	0.127	0.154	0.125	0.126
HSV16	0.076	0.040	0.109	0.127	0.188	0.120	0.111	0.105	0.131	0.193	0.166	0.196	0.180	0.175
HSV17	0.158	0.152	0.065	0.086	0.050	0.067	0.095	0.099	0.086	0.115	0.072	0.112	0.133	0.128
HSV18	0.265	0.240	0.230	0.246	0.263	0.267	0.261	0.278	0.308	0.347	0.328	0.341	0.357	0.356
HSV19	0.352	0.163	0.136	0.172	0.180	0.173	0.177	0.188	0.205	0.217	0.249	0.256	0.319	0.322
HSV20	0.047	0.182	0.288	0.329	0.272	0.159	0.164	0.168	0.178	0.192	0.177	0.208	0.179	0.196
HSV21	0.040	0.024	0.102	0.124	0.178	0.172	0.159	0.149	0.170	0.187	0.143	0.135	0.148	0.156
HSV22	0.177	0.251	0.194	0.314	0.416	0.457	0.442	0.437	0.404	0.385	0.366	0.340	0.287	0.320
HSV23	0.252	0.248	0.226	0.253	0.206	0.187	0.187	0.283	0.333	0.300	0.295	0.272	0.292	0.276
HSV24	0.753	0.763	0.746	0.864	0.857	0.854	0.830	0.773	0.762	0.743	0.776	0.744	0.776	0.775
HSV25	0.141	0.581	0.313	0.274	0.242	0.227	0.226	0.212	0.206	0.212	0.245	0.226	0.223	0.222
HSV26	0.284	0.311	0.382	0.453	0.397	0.353	0.327	0.324	0.326	0.305	0.355	0.339	0.382	0.361
HSV27	0.434	0.212	0.118	0.125	0.146	0.125	0.135	0.144	0.177	0.180	0.196	0.199	0.342	0.350
HSV28	0.087	0.244	0.623	0.677	0.574	0.436	0.390	0.389	0.416	0.437	0.471	0.497	0.553	0.521
HSV29	0.131	0.121	0.095	0.101	0.148	0.172	0.168	0.175	0.166	0.173	0.146	0.174	0.144	0.150
HSV30	0.608	0.624	0.607	0.722	0.711	0.723	0.719	0.694	0.697	0.663	0.708	0.677	0.673	0.671
HSV31	0.075	0.132	0.158	0.151	0.216	0.320	0.311	0.323	0.325	0.312	0.290	0.283	0.290	0.280
HSV32		0.135	0.148	0.057	0.055	0.062	0.090	0.077	0.071	0.061	0.033	0.055	0.142	0.141
HSV33		0.614	0.323	0.327	0.242	0.249	0.256	0.188	0.185	0.173	0.247	0.215	0.155	0.157
HSV34			0.493	0.539	0.466	0.350	0.318	0.314	0.330	0.325	0.373	0.401	0.406	0.393
HSV35			0.201	0.259	0.255	0.247	0.246	0.169	0.203	0.205	0.202	0.171	0.192	0.191
HSV36				0.194	0.183	0.181	0.188	0.169	0.162	0.164	0.180	0.211	0.186	0.189
HSV37				0.301	0.342	0.329	0.308	0.305	0.287	0.331	0.305	0.328	0.249	0.236
HSV38				0.380	0.400	0.399	0.371	0.378	0.386	0.409	0.403	0.398	0.392	0.387
HSV39				0.231	0.197	0.181	0.191	0.209	0.189	0.178	0.156	0.159	0.170	0.180
HSV48							0.396	0.392	0.409	0.400	0.411	0.441	0.458	0.466
HSV49							0.197	0.191	0.192	0.207	0.198	0.204	0.215	0.215
HSV50							0.177	0.229	0.247	0.214	0.233	0.201	0.226	0.223
HSV51							0.048	0.047	0.032	0.033	0.024	0.050	0.104	0.109
HSV52								0.302	0.366	0.322	0.317	0.292	0.293	0.283
HSV53								0.386	0.329	0.309	0.332	0.304	0.333	0.334
HSV54									0.246	0.267	0.217	0.238	0.251	0.254
HSV55									0.119	0.135	0.102	0.153	0.115	0.119
HSV56									0.119	0.130	0.115	0.158	0.138	0.141
HSV57									0.304	0.284	0.312	0.298	0.271	0.283
HSV58										0.230	0.212	0.233	0.185	0.167
HSV59										0.292	0.231	0.243	0.256	0.251
HSV60										0.105	0.137	0.118	0.198	0.201
HSV61										0.148	0.105	0.149	0.205	0.189
HSV62											0.085	0.104	0.118	0.103
HSV63											0.150	0.180	0.198	0.193
HSV64											0.287	0.295	0.333	0.322
HSV65											0.237	0.247	0.186	0.185
HSV66												0.216	0.234	0.234
HSV67												0.246	0.218	0.216
HSV68													0.635	0.603
HSV69													0.419	0.406
HSV70														0.300
HSV71														0.148

**Table 6:** Results of IBS-central method starting from a RP of 30 members. Blue denotes the central animal being the most similar in genetic composition to all other animals. Red denotes the border defined by an HWV animal and the HSVs farthest from the central animal. Due to space limitations of the tables, not all animals (rows) and addition steps (columns) are presented here; the full dataset is included in Supplementary Table S3.

Animal Number	30	32	34	38	40	46	50	52	56	60	64	66	68	70
ID	Distance to Central Animal													
HWV1	1.000	1.000	1.000	1.000	1.000	1.000	1.000	1.000	1.000	1.000	1.000	1.000	1.000	1.000
HSV2	0.112	0.113	0.112	0.115	0.118	0.124	0.131	0.126	0.116	0.119	0.123	0.124	0.121	0.118
HSV3	0.069	0.053	0.048	0.065	0.065	0.083	0.085	0.082	0.082	0.086	0.094	0.095	0.096	0.094
HSV4	0.000	0.000	0.000	0.000	0.000	0.000	0.005	0.005	0.000	0.000	0.013	0.012	0.015	0.017
HSV5	0.047	0.039	0.033	0.050	0.051	0.063	0.069	0.070	0.069	0.072	0.078	0.079	0.079	0.081
HSV6	0.194	0.201	0.196	0.202	0.200	0.198	0.205	0.203	0.207	0.205	0.207	0.208	0.199	0.198
HSV7	0.204	0.206	0.206	0.185	0.188	0.179	0.181	0.179	0.174	0.173	0.178	0.181	0.180	0.181
HSV8	0.250	0.249	0.249	0.248	0.240	0.247	0.251	0.254	0.248	0.233	0.233	0.233	0.229	0.228
HSV9	0.046	0.050	0.049	0.051	0.049	0.067	0.073	0.071	0.052	0.053	0.062	0.063	0.060	0.053
HSV10	0.197	0.194	0.189	0.180	0.176	0.184	0.188	0.192	0.185	0.186	0.186	0.184	0.178	0.180
HSV11	0.170	0.172	0.160	0.166	0.167	0.174	0.162	0.163	0.164	0.165	0.172	0.178	0.178	0.180
HSV12	0.237	0.233	0.216	0.210	0.204	0.200	0.203	0.202	0.200	0.203	0.200	0.200	0.193	0.189
HSV13	0.050	0.050	0.043	0.040	0.026	0.029	0.034	0.035	0.037	0.038	0.042	0.040	0.039	0.035
HSV14	0.054	0.049	0.044	0.032	0.030	0.041	0.044	0.043	0.036	0.037	0.038	0.039	0.035	0.034
HSV15	0.072	0.070	0.067	0.073	0.070	0.078	0.080	0.080	0.076	0.075	0.078	0.078	0.074	0.074
HSV16	0.141	0.143	0.137	0.122	0.113	0.122	0.122	0.122	0.122	0.119	0.124	0.126	0.123	0.123
HSV17	0.212	0.209	0.208	0.204	0.199	0.215	0.218	0.218	0.212	0.188	0.200	0.200	0.197	0.196
HSV18	0.262	0.262	0.257	0.247	0.237	0.246	0.245	0.248	0.251	0.244	0.250	0.254	0.244	0.245
HSV19	0.075	0.069	0.060	0.079	0.078	0.086	0.094	0.092	0.090	0.092	0.096	0.095	0.096	0.095
HSV20	0.199	0.209	0.194	0.194	0.189	0.188	0.186	0.187	0.176	0.179	0.184	0.184	0.184	0.181
HSV21	0.127	0.120	0.090	0.081	0.082	0.083	0.082	0.083	0.084	0.089	0.087	0.086	0.081	0.081
HSV22	0.133	0.126	0.131	0.112	0.088	0.100	0.111	0.114	0.117	0.117	0.124	0.123	0.119	0.112
HSV23	0.374	0.379	0.381	0.386	0.385	0.393	0.392	0.369	0.366	0.370	0.376	0.380	0.375	0.373
HSV24	0.600	0.607	0.599	0.604	0.607	0.624	0.622	0.613	0.618	0.621	0.623	0.624	0.623	0.622
HSV25	0.261	0.218	0.222	0.225	0.224	0.239	0.242	0.242	0.244	0.237	0.238	0.235	0.233	0.230
HSV26	0.270	0.275	0.263	0.270	0.265	0.277	0.279	0.272	0.279	0.283	0.290	0.293	0.288	0.286
HSV27	0.124	0.126	0.117	0.121	0.125	0.127	0.131	0.133	0.127	0.124	0.135	0.136	0.142	0.143
HSV28	0.306	0.309	0.270	0.283	0.272	0.270	0.270	0.271	0.272	0.271	0.277	0.281	0.272	0.270
HSV29	0.164	0.162	0.162	0.160	0.146	0.155	0.161	0.162	0.151	0.150	0.152	0.149	0.149	0.145
HSV30	0.425	0.421	0.415	0.422	0.424	0.440	0.445	0.431	0.440	0.444	0.448	0.448	0.449	0.451
HSV31	0.109	0.112	0.116	0.109	0.101	0.095	0.112	0.112	0.107	0.101	0.110	0.106	0.108	0.106
HSV32		0.202	0.194	0.197	0.195	0.197	0.178	0.178	0.179	0.183	0.173	0.173	0.174	0.175
HSV33		0.249	0.246	0.250	0.244	0.262	0.269	0.271	0.272	0.268	0.271	0.270	0.266	0.262
HSV34			0.133	0.145	0.143	0.133	0.137	0.136	0.135	0.138	0.140	0.143	0.140	0.140
HSV35			0.223	0.213	0.215	0.226	0.229	0.233	0.229	0.234	0.240	0.237	0.230	0.229
HSV36				0.105	0.107	0.108	0.112	0.114	0.107	0.111	0.111	0.109	0.109	0.106
HSV37				0.185	0.174	0.182	0.190	0.193	0.190	0.181	0.191	0.194	0.192	0.194
HSV38				0.380	0.384	0.393	0.392	0.394	0.398	0.395	0.401	0.403	0.402	0.405
HSV39				0.138	0.138	0.147	0.160	0.163	0.163	0.160	0.171	0.170	0.162	0.159
HSV48							0.214	0.219	0.217	0.223	0.228	0.233	0.231	0.231
HSV49							0.231	0.229	0.228	0.220	0.222	0.216	0.214	0.210
HSV50							0.287	0.276	0.278	0.280	0.283	0.286	0.281	0.279
HSV51							0.000	0.000	0.002	0.004	0.000	0.000	0.000	0.000
HSV52								0.216	0.214	0.218	0.226	0.227	0.225	0.223
HSV53								0.239	0.233	0.232	0.232	0.232	0.233	0.232
HSV54									0.241	0.239	0.240	0.240	0.236	0.233
HSV55									0.112	0.113	0.119	0.122	0.118	0.119
HSV56									0.059	0.060	0.063	0.062	0.062	0.063
HSV57									0.124	0.121	0.126	0.122	0.123	0.116
HSV58										0.132	0.138	0.139	0.139	0.142
HSV59										0.193	0.197	0.198	0.196	0.195
HSV60										0.164	0.170	0.169	0.169	0.168
HSV61										0.166	0.177	0.180	0.175	0.175
HSV62											0.088	0.086	0.087	0.088
HSV63											0.140	0.141	0.129	0.127
HSV64											0.255	0.257	0.250	0.249
HSV65											0.159	0.153	0.150	0.150
HSV66												0.111	0.114	0.113
HSV67												0.014	0.013	0.011
HSV68													0.470	0.468
HSV69													0.267	0.268
HSV70														0.138
HSV71														0.172

**Table 7:** Standardised values of artificially admixed animals calculated by PCA-distance and IBS-central methods.

	PCA-Distance				IBS-Central			
	lowP		highP		lowP		highP	
% HWV	HSV38	HSV68	HSV45	HSV67	HSV38	HSV68	HSV45	HSV67
	Distance to HSV Mean				Distance to Central Animal			
0	0.369	0.610	0.138	0.263	0.405	0.468	0.097	0.110
10	0.745	0.870	0.551	0.229	0.476	0.585	0.235	0.169
20	0.768	0.848	0.461	0.280	0.500	0.564	0.327	0.276
30	0.777	0.881	0.523	0.429	0.542	0.639	0.392	0.363
40	0.810	0.916	0.625	0.531	0.616	0.722	0.471	0.463
50	0.875	0.977	0.797	0.714	0.688	0.823	0.595	0.581
60	0.910	1.024	0.875	0.807	0.765	0.948	0.741	0.676
70	0.953	1.051	0.932	0.892	0.842	1.000	0.836	0.784
80	0.958	1.063	0.958	0.935	0.859	1.000	0.890	0.842
90	0.967	0.979	0.961	0.955	0.892	0.925	0.886	0.889

## Data Availability

The data presented in this study are available on request from the first and the corresponding author, and with the permission of Hungarian kennel clubs of the corresponding breed.

## Supplementary Materials

The following supporting information can be downloaded at: https://www.mdpi.com/article/10.3390/genes13112022/s1, Figure S1: Oscillations of standardised values obtained from the PCA-distance method; Figure S2: Oscillations of standardised values obtained from the IBS-central method; Table S1: List of 924 HSV-enhanced set; Table S2: Examples for calculation of PCA-distance and IBS-central methods and PCA coordinates of Figure 3; Table S3: Results of PCA-distance and IBS-central methods.; Table S4: Ordered and colour-coded results of PCA-distance and IBS-central methods.
